# A major QTL on chromosome 7HS controls the response of barley seedling to salt stress in the Nure × Tremois population

**DOI:** 10.1186/s12863-017-0545-z

**Published:** 2017-08-22

**Authors:** Wentao Xue, Jun Yan, Gang Zhao, Yan Jiang, Jianping Cheng, Luigi Cattivelli, Alessandro Tondelli

**Affiliations:** 1 College of Life Sciences, Guizhou University, Guiyang, Guizhou 550025 China; 2 School of Pharmacy and Bioengineering, Chengdu University, Chengdu, Sichuan 610106 China; 3 College of Agriculture, Guizhou University, Guiyang, Guizhou 550025 China; 4 CREA, Research Centre for Genomics and Bioinformatics, 29017 Fiorenzuola d’Arda, Italy

**Keywords:** Barley, Salt tolerance, Seedling, QTL

## Abstract

**Background:**

Seedling establishment is a crucial and vulnerable stage in the crop life cycle which determines further plant growth. While many studies are available on salt tolerance at the vegetative stage, the mechanisms and genetic bases of salt tolerance during seedling establishment have been poorly investigated. Here, a novel and accurate phenotyping protocol was applied to characterize the response of seedlings to salt stress in two barley cultivars (Nure and Tremois) and their double-haploid population.

**Results:**

The combined phenotypic data and existing genetic map led to the identification of a new major QTL for root elongation under salt stress on chromosome 7HS, with the parent Nure carrying the favourable allele. Gene-based markers were developed from the rice syntenic genomic region to restrict the QTL interval to Bin2.1 of barley chromosome 7HS. Furthermore, doubled haploid lines with contrasting responses to salt stress revealed different root morphological responses to stress, with the susceptible genotypes exhibiting an overall reduction in root length and volume but an increase in root diameter and root hair density.

**Conclusions:**

Salt tolerance at the seedling stage was studied in barley through a comprehensive phenotyping protocol that allowed the detection of a new major QTL on chromosome 7HS.

**Electronic supplementary material:**

The online version of this article (doi:10.1186/s12863-017-0545-z) contains supplementary material, which is available to authorized users.

## Background

Salinity is a serious threat to agricultural crop productivity due to adverse effects on germination rate, seedling establishment [1], plant vigour and crop yield [2]. The limited salt tolerance of major crops renders agriculture vulnerable in many regions worldwide, especially in arid and semi-arid areas in Australasia, North–Central Asia and South America [3], where irrigation systems are prone to accelerating soil salinization [4]. Therefore, improving crop salt tolerance is a priority for plant breeding [5, 6]. Different phenotyping procedures have been developed to search for halophytes [7–9] or mutant lines [10, 11] that are better adapted to high salt conditions, or to scrutinize the natural variation in salt tolerance within germplasm collections of relevant crops [12, 13]. These works have led to the identification of salt-responsive genes in both crop and model plants [14–17].

Plants have developed different mechanisms to deal with saline conditions, for instance, by excluding Na^+^/Cl^−^ from uptake, controlling xylem Na^+^/Cl^−^ loading and/or its retrieval from the shoot, efficient vacuolar Na^+^/Cl^−^ sequestration, cytosolic K^+^/Cl^−^ homeostasis and retention in root and mesophyll cells, efficient osmotic adjustment, and reactive oxygen species (ROS) detoxification [15]. Of the cereal crops, barley is the most salt-tolerant, as it can tolerate NaCl solutions of up to 250 mM [18], with cultivar Numar being able to grow in 320 mM NaCl solutions [7, 19]. The genetic basis of barley salt tolerance has been investigated in depth [7, 20], with a number of quantitative trait loci (QTLs) having been mapped on different chromosomes [21, 22]. Nevertheless, most of these studies focus on the vegetative stages, with few works dealing with the initial and vulnerable seedling phase [23].

Seedling establishment is greatly affected by salinity [24], resulting in poor and delayed seedling emergence [25]. A few studies have compared the effects of salt on plant growth at different developmental stages [26–30], and the results suggested that the QTLs controlling salt tolerance during seedling establishment differ from those controlling the same response in older plants [27, 31]. In barley, two major QTLs for salt tolerance at the seedling stage have been identified on chromosome 5H in the Steptoe × Morex (SM) and Oregon Wolfe Barley (OWB) mapping populations, linked with the restriction fragment length polymorphism (*RFLP*) markers *ABC324* (Bin7)[31] and *ABC302* (Bin8)[23], respectively. In the same region, a QTL for seed pre-harvest sprouting and dormancy was also detected, suggesting a general role in seed germination rather than a specific response to salt stress for this locus [32, 33]. Another study based on the OWB population mapped a major QTL controlling the response of 10-day-old seedlings to salt stress to chromosome 7H (Bin7), with *Bmag0303a* and *Nud* as flanking markers [34].

Phenotyping of seedlings under salt stress suffers from methodological limitations [35]. For small seeds, such as those of *Arabidopsis*, culture media with different salt concentrations are commonly used to germinate the seeds vertically on a horizontal plate [36–38]. Similar methodologies are less appropriate for larger cereal seeds, due to space limitations and problems observing the intertwined roots and shoots; cereal salt tolerance at the seedling stage is therefore usually assessed using a descriptive score [23, 31]. For example, classification of barley seedlings from two mapping populations was based on a scale of 1 to 5 according to the observed levels of leaf injury after salt-stress treatment [31]. Tolerance indices or scores have also been developed to calculate seedling length or weight relative to control condition [12]. Although QTLs have been frequently mapped with these datasets, a mathematically comprehensive depiction of the dynamic response of seedlings to salinity is lacking. These limitations might influence mapping accuracy, and subsequently affect both genetic analyses and breeding efforts toward enhancing salt tolerance [35]. The mathematical model of the sigmoidal Hill equation has been applied to a description of seed dormancy and germination, as well as germination under salt stress [39], and key parameters of the derived simulated curves have been extracted for further genetic analyses [40]. To date, similar mathematical models have not been adopted for barley seedling growth under saline conditions.

The barley Nure × Tremois (NT) double-haploid (DH) population has been successfully exploited for mapping complex traits, including frost tolerance [41, 42], yield adaptation [43, 44] and malting quality [45]. Given that Nure and Tremois differ in terms of seedling growth capacity under salt conditions, the NT population was employed to search for the loci determining salt tolerance at the seedling stage. The genotypes were evaluated with a novel vertical germination system to describe dynamic seedling curves in response to multiple NaCl treatments, and the key parameters of the seedling curves were extracted for QTL mapping. Genetic analyses identified a major QTL on chromosome 7HS which was further characterized by exploiting its syntenic relationship with rice chromosome 6S. Furthermore, the stress-induced morphological responses (SIMRs) of barley seedlings to different salt concentrations were investigated in DH lines displaying contrasting responses to salt stress.

## Methods

### Plant materials

We used 118 DH barley lines obtained from a cross between the Italian two-row winter feeding cv. Nure [(Fior 40 × Alpha) × Baraka] and the French two-row spring malting cv. Tremois [(Dram **×** Aramir) **×** Berac]. Seeds of the whole DH population were propagated in Fiorenzuola, Italy, in the 2007–2008 growing season and stored under cool (+7 °C) and dry (40% relative humidity) conditions. Maximum germination percentage (*G*_*max*_), tested for each DH line before the experiment, was >90%.

### Vertical seed germination under salt stress

Plates composed of two plastic sheets (A4 size) and two filter papers (50 × 50 cm, Delchimica, Italy) were used to clamp seeds for germination (Additional file 1-A); the filter papers served as medium, taking up and maintaining the water or salt solutions. The plates were pre-soaked in water or salt solutions before seed sowing, and then placed vertically in plastic boxes, with the papers in constant contact with the water or salt solution (Additional file 1-B). Each box contained 20 plates with seeds plus two empty plates in the first and last position, to minimize spatial differences in evaporation (Additional file 1-C). A plastic sheet was used to keep the plates vertical in the box (Additional file 1-D) and the boxes were covered to reduce evaporation (Additional file 1-B). Seeds were surface-sterilized with 3% (v/v) H_2_O_2_ for 30 min before sowing, then rinsed three times with water. For each DH line, 10 seeds were placed in the hood of the filter paper (Additional file 1-E) for germination. Different levels of salt stress were simulated independently using 80, 160, 240, 320 or 400 mM NaCl solutions, in parallel with a control solution (no salt added). The purified water for the control and salt solutions was generated by the Pure/Ultrapure Water System (Millipore), with an electrical conductivity of 5.39 μS/cm.

To prevent the formation of salt gradients during germination and seedling growth, the solutions were replaced daily and all plates were soaked daily in their respective solutions. Seeds of the whole DH population were germinated using a randomized complete block design with three replicates for each salt-stress treatment, and six replicates for control condition. After 7 days in a growth chamber at 20 °C without light, the filter papers with seedlings were placed under a camera at a fixed distance of 35 cm, and photographed (180 × 180 dpi) (Additional file 2). A reference ruler was also photographed independently in each germinating set.

To prevent potential effects of seed source on seedling growth, 20 DH lines characterized by contrasting seedling responses to salt stress were propagated in a different environment (Reggio Emilia, Italy, during the 2012–2013 growing season) and used to validate the results. Tolerant and susceptible groups were defined, with NT030, NT022, NT057, NT071, NT078, NT086, NT115, NT117, NT124 and NT141 belonging to the tolerant group and NT004, NT043, NT051, NT063, NT075, NT084, NT085, NT093, NT098 and NT131 belonging to the susceptible group. All 20 lines were germinated as previously described with three replicates.

Finally, two DH lines showing contrasting behaviour (NT030 and NT131) were characterized by the same procedure for 14 days: 7 days of salt-stress treatment followed by 7 days of non-stressed recovery under a 14-h photoperiod. The seedlings were photographed daily.

### Image analysis

All images were analysed with the free ImageJ software v.1.49 (http://imagej.nih.gov/ij/). First, the “Straight Lines” function was used to calculate the number of pixels for 1 cm on the reference ruler (85 pixel/cm in our case). The function “Segmented Lines” was then applied to measure the length of the seminal roots and shoots (RL and SL, respectively), with RL representing root depth. For each line and replicate, independent measurements from three representative plants were taken (Additional file 2) and averaged. In total, nine individual measurements were taken for each genotype from three images.

In addition, the roots of 12 DH lines (from the 20 DH lines selected for the validation experiment) were also scanned independently with an EPSON Expression 10000XL scanner and the images (400 dpi) were analysed with WinRHIZO software [46] to measure the following traits: total root length (TRL, the total length of each seminal root), average seminal root diameter (RD), total seminal root volume (RV) and total number of seminal root tips (RT).

### Modelling and statistical analysis

**Fig. 1:**
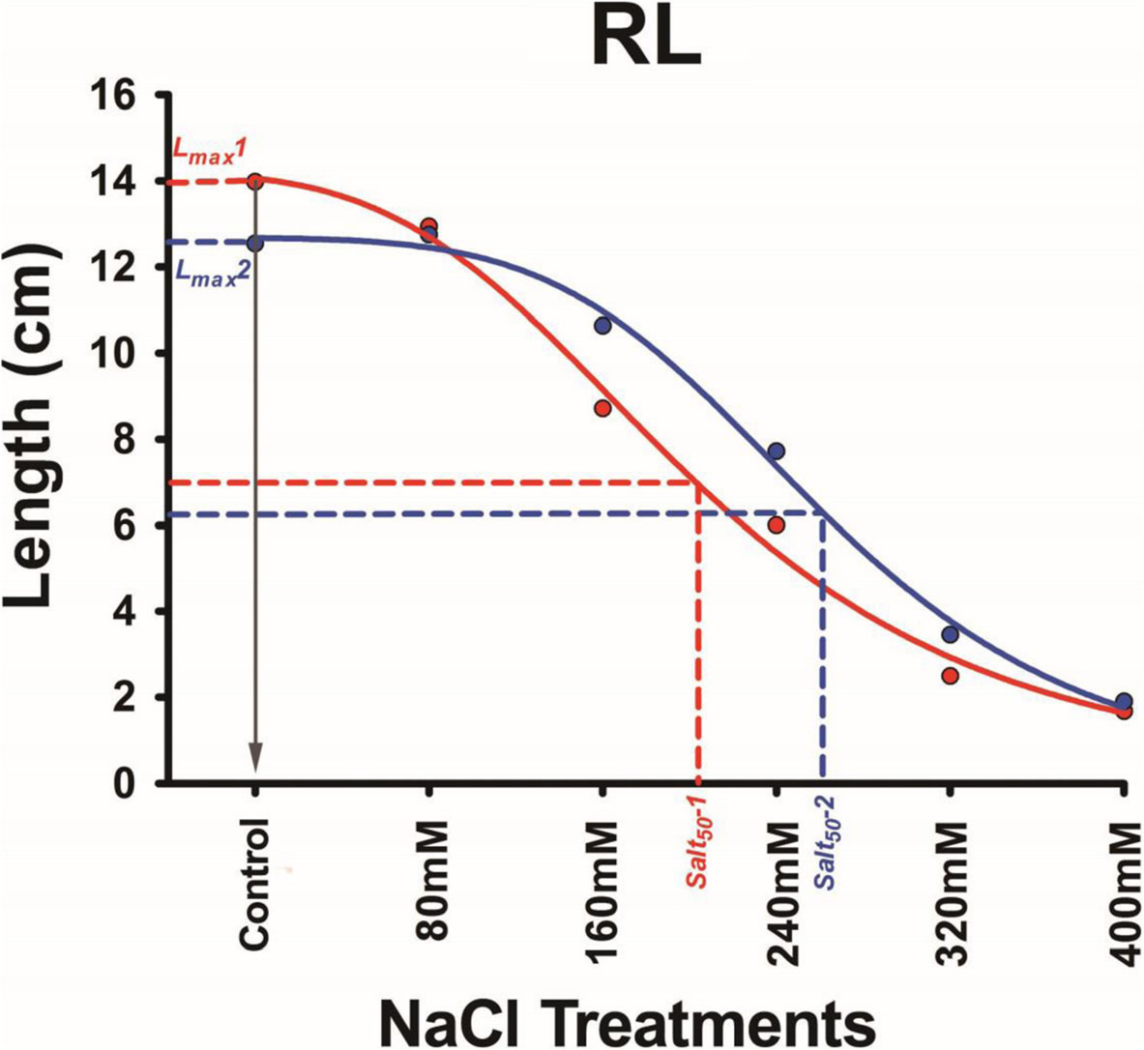
Modelling the dynamic response of barley seedling length to salt stress. Each point represents the root length (RL) after 7 days of germination in different NaCl treatments. *L*_*max*_ was estimated based on the Hill three-parameter equation where *x* is the control NaCl concentration (0.001 mM). *Salt*_*50*_ was calculated based on a 50% reduction of *L*_*max*_. *Red* and *blue lines* refer to DH lines NT118 and NT123, respectively

Sigmaplot 12.0 (Systat) was applied to each line to draw a scatter graph with seedling length on the *y* axis and NaCl concentration on the *x* axis, as illustrated in Fig. 1 for tolerant and susceptible DH lines (NT123 and NT118, respectively) characterized by different RLs under control condition and different responses to salt stress. The control solution was approximated to 0.001 mM NaCl since water electrical conductivity (5.39 μS/cm) was equivalent to 589.7 nM NaCl (1 μS/cm represents 0.64 mg of NaCl per 1 L of water). Therefore, seedling lengths were plotted against concentrations of 0.001, 80, 160, 240, 320 and 400 mM NaCl (water contribution of 0.001 mM was not relevant for the salt treatments). For each line, the six points were used to fit curves following the three-parameter Hill function:

$$ y=\frac{ax^b}{c^b+{x}^b} $$

where variables *a*, *b* and *c* were estimated by Sigmaplot 12.0 and defined according to Hill modelling for seed germination [39, 40]; *a* is a limit value for the maximum seedling length (*L*_*max*_), *b* controls the shape and steepness of the simulated curve, and *c* is the half-maximal activation level of the curve. *L*_*max*_ was calculated for each line by solving the Hill equation as follows:

when *x* = 0.001 mM, *L*_*max*_ $=\frac{a\times {(0.001)}^b}{c^b+{(0.001)}^b}$

*Salt*_*50*_ represents the NaCl concentration leading to a half-reduction in seedling *L*_*max*_ and it was calculated as follows:

*Salt*_*50*_ (mM) =$$ \sqrt[b]{\frac{\left({L}_{max}\times 0.5\right)\times {c}^b}{a-\left({L}_{max}\times 0.5\right)}} $$

Similarly, the parameters *Salt*_*80*_, *Salt*_*70*_, *Salt*_*60*_, *Salt*_*40*_, *Salt*_*30*_ and *Salt*_*20*_ were estimated for 80, 70, 60, 40, 30 and 20% reductions of seedling *L*_*max*_. All parameters (*L*_*max*_ and *Salt*_*80*_ *–Salt*_*20*_) extracted above were defined as response parameters (REPs).

Overall, 18 phenotypic parameters, including seedling length under control condition and the REPs, were used for QTL analysis (Table 1). *L*_*max*_ was regarded as a non-stressed seedling trait because of its strong correlation with seedling length under control condition (*R* ^2^ > 0.85). Heritability (*H* ^*2*^) of the REPs was calculated as previously described [47]:

$$ {H}^2=\frac{\sigma^2g}{\sigma^2g+{\sigma}^2e} $$

where *σ* ^2^ *e* is the residual variance component and *σ* ^*2*^ *g* is the genotypic variance component.

**Table 1:** List of phenotypic traits evaluated in the NT barley population

Class of traits	Measurements and Calculations	Unit	Traits
Seedling length	Seedling length without stress	cm	RL-Control, SL-Control
	*L*_*max*_ of simulated curves	cm	RL-*L*_*max*_, SL-*L*_*max*_
Response parameter	*Salt*_*80–20*_ for root length under salt stress	mM	RL-*Salt*_*80*_, RL-*Salt*_*70*_, RL-*Salt*_*60*_, RL-*Salt*_*50*_, RL-*Salt*_*40*_, RL-*Salt*_*30*_, RL-*Salt*_*20*_
	*Salt*_*80–20*_ for shoot length under salt stress	mM	SL-*Salt*_*80*_, SL-*Salt*_*70*_, SL-*Salt*_*60*_, SL-*Salt*_*50*_, SL-*Salt*_*40*_, SL-*Salt*_*30*_, SL-*Salt*_*20*_

For the validation experiments on 20 selected lines, two-way ANOVA was conducted using STATISTICA 7, taking environment and genotype as factors.

### Development of molecular markers

The molecular characterization of the NT DH lines and the development of the NT genetic linkage map were as described in previous studies [43, 44]. In the present work, 25 new molecular markers were added to the existing genetic map (see Additional file 3 for primer information). Simple sequence repeat (*SSR*) markers were selected according to the barley consensus *SSR* map [48]; markers on chromosome 5H were developed from the barley transcript map [49]. To increase marker density on chromosome 7HS, *iSelect* single nucleotide polymorphism (*SNP*)-based markers were selected [50]. In addition, several genes in this region were identified by exploiting syntenic information from the rice genome (chromosome 6S, http://rice.plantbiology.msu.edu/), and the corresponding contigs from the reference Morex genome (https://ics.hutton.ac.uk/morexGenes/) were used as templates for primer design. Sequence-tagged sites (*STS*), cleaved amplified polymorphic sequences (*CAPS*) and derived-*CAPS* (*dCAPS*) markers were developed from nucleotide polymorphisms after PCR amplification and sequencing of the parents Nure and Tremois. DNA amplifications from the whole DH population were carried out in 10 μL reaction mixture containing 10 ng template DNA, 0.25 μM of each primer, 1.5 mM MgCl_2_, 0.25 mM of each deoxynucleoside triphosphate, and 1.5 units of Taq DNA polymerase (Tiangen, China). Three different PCR amplification protocols were adopted, as described in Additional file 3. The new linkage map was calculated by JoinMap 4 (https://www.kyazma.nl/index.php/JoinMap).

### QTL analysis

QTL analysis was performed with the software MapQTL 6.0 (https://www.kyazma.nl/index.php/mc.MapQTL). The interval mapping (IM) function was first applied to detect significant QTLs passing a logarithm of odds (LOD) threshold of 3. The peak markers were then selected as co-factors for composite interval mapping (CIM). Linkage maps and QTLs were graphically represented with MapChart 2.2 [51].

## Results

### Dynamic response of barley seedlings to salt stress

**Fig. 2:**
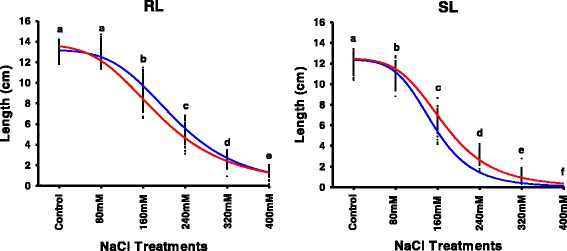
RL and SL measured for the Nure and Tremois parents and the NT population under different NaCl treatments. Each point represents the length of the root or shoot for a single genotype, after 7 days of germination in different salt treatments. Blue and red curves show the responses of the parental genotypes Nure and Tremois, respectively. Letters on the graph denote statistically significant differences between salt treatments (Tukey HSD, *p < 0.05*)

Preliminary experiments indicated that the seedling growth capacities of Nure and Tremois differ when exposed to salinity. This, along with the availability of a DH population derived from a cross between these two cultivars allowed a detailed investigation of the genetic basis controlling seedling growth under salt stress. The dynamic response of seedlings to different salt concentrations is presented in Fig. 2 for the parents and the whole NT population. Notably, Nure and Tremois showed contrasting responses for both RL and SL curves (Fig. 2), with Nure having a higher RL and lower SL than the Tremois, suggesting different genetic controls for root and shoot growth under salt conditions. Moreover, comparisons of the individual treatments revealed significant growth reductions for both roots and shoots with increasing salt concentration, except for RL at 80 mM NaCl which was not significantly different from the control (Fig. 2). Most of the DH lines were able to produce seminal roots in 400 mM NaCl, but rarely produced shoots. The REPs *L*_*max*_, *Salt*_*80*_, *Salt*_*70*_, *Salt*_*60*_, *Salt*_*50*_, *Salt*_*40*_, *Salt*_*30*_ and *Salt*_*20*_ were extracted from the simulated curves, as illustrated in Fig. 1, and their data range, average values and *H* ^*2*^ values are reported in Additional file 4, together with the values from the parent lines. Consistent with the above results, Nure showed higher values than Tremois for RL-REPs, but lower values for SL-REPs. Higher *H* ^*2*^ values were observed for RL-REPs vs. SL-REPs, with average values of 0.70 and 0.63, respectively, suggesting a strong genetic basis.

### QTL analysis of seedling growth under control and saline conditions

**Fig. 3:**
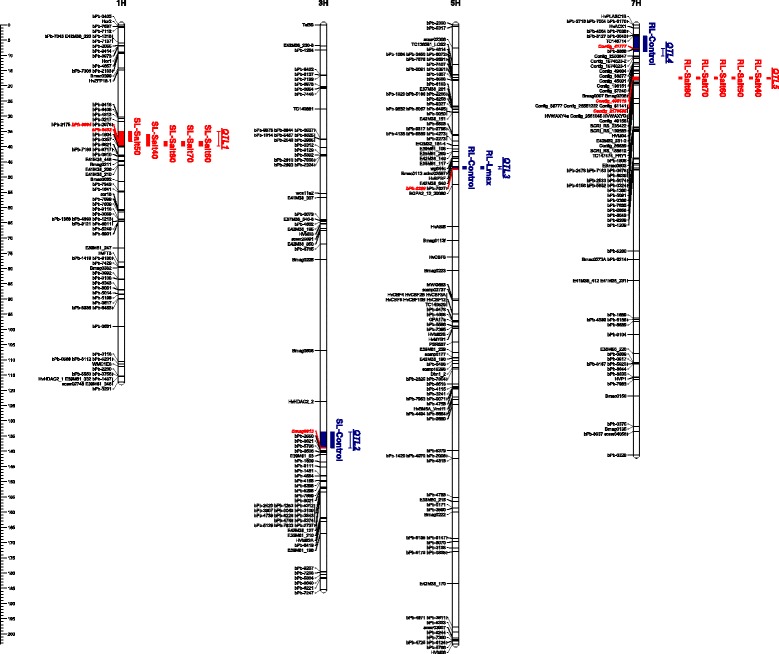
QTLs detected in NT barley population for the seedling response to salt stress*.* QTLs are drawn based on the results from CIM. QTLs controlling seedling growth under control condition are labelled in *blue*; those controlling seedling growth in response to salinity are in *red*. The peak markers for each QTL are indicated in *red*

Of the 18 phenotypic parameters considered for QTL analysis, 13 traits allowed the identification of 5 QTL regions on 4 barley chromosomes. The results are presented in Table 2 and Fig. 3, where the QTLs detected for non-stressed (control) and salt-stressed seedlings are given in blue and red colors, respectively. The analysis of seedling growth under control condition identified three main significant regions on chromosomes 3H (SL-Control), 5H (RL-Control and RL-*L*_*max*_) and 7H (RL-Control). The QTL analysis for salt-stressed seedlings highlighted a region for shoot-related traits on chromosomes 1H and another region for root-related traits on chromosome 7H (Fig. 3). No overlapping QTLs were observed between RL and SL, or between non-stressed and salt-treated seedlings, even though the RL-Control QTL (QTL4) on chromosome 7H mapped in the proximity of the main region (QTL5) controlling root elongation for salt-stressed seedlings.

**Table 2:** QTLs associated with the response of barley seedlings to salt stress in the NT population*.* Results from CIM are presented

Chromosome	Name	Trait	QTL interval^a^	Peak marker	Position (cM)	LOD	*R* ^*2*^ (%)	Additive effect^b^
1H	QTL1	SL-*Salt*_*50*_	35.0-38.3	*bPb-8884*	38.3	3.40	12.7	−4.42
		SL-*Salt*_*40*_	36.0–38.3	*bPb-8884*	38.3	3.21	11.6	−4.46
		SL-*Salt*_*80*_	38.3–39.8	*bPb-0482*	39.8	3.31	12.4	−4.24
		SL-*Salt*_*70*_	38.3–39.8	*bPb-0482*	39.8	3.46	12.9	−4.34
		SL-*Salt*_*60*_	38.3–39.8	*bPb-0482*	39.8	3.49	13.0	−4.40
3H	QTL2	SL-Control	133.7–138.9	*Bmag0013*	138.9	3.49	11.6	0.22
5H	QTL3	RL-Control	46.5–47.4	*bPb-6260*	47.4	3.91	10.9	−0.19
		RL-*L*_*max*_	46.5–47.4	*bPb-6260*	47.4	4.29	15.8	−0.20
7H	QTL4	RL-Control	3.7–8.8	*Contig_41777*	7.8	4.45	16.3	0.23
	QTL5	RL-*Salt*_*80*_	17.2–17.8	*Contig_2179585*	17.8	15.05	45.3	12.99
		RL-*Salt*_*70*_	17.2–17.8	*Contig_2179585*	17.8	14.94	45.0	12.74
		RL-*Salt*_*60*_	17.2–17.8	*Contig_405119*	17.2	14.58	44.2	12.27
		RL-*Salt*_*50*_	17.2–17.8	*Contig_405119*	17.2	13.77	42.4	11.67
		RL-*Salt*_*40*_	17.2–17.8	*Contig_405119*	17.2	12.19	38.6	10.82

^a^QTL intervals are defined according to LOD threshold >3

^b^Positive sign indicates that Nure allele increases phenotypic value

QTL1 was associated with five SL-REPs, and spanned a region between 35.0 and 39.8 cM on chromosome 1HS (Table 2). BLAST analyses of the peak *DA*r*T* markers *bPb-8884* and *bPb-0482* revealed similarities with *Contig_56662* and *Contig_1602477* at position 48–50 cM on the Morex × Barke POPSEQ map [52]. The favourable allele (i.e., longer shoot under salt conditions) at this QTL was contributed by Tremois. QTL2 mapped to a region between 133.7 and 138.9 cM on chromosome 3HL responsible for SL under control condition, with Nure carrying the favourable allele. QTL3 for RL-Control (favourable alleles contributed by Tremois) was defined by the peak marker *bPb-6260*, which matched *Contig_1570386* at position 43.76 cM on chromosome 5HL of the OWB POPSEQ map [52]. Notably, QTL3 also mapped close to a major QTL previously reported for salt tolerance of 10-day-old seedlings in the OWB mapping population (peak marker *GBS0318* located at 50.44 cM, Witzel et al. 2010 [23]). No polymorphisms were detected for *GBS0318* between the Nure and Tremois parents, while the close *SNP* marker, *BOPA2_12_30080* (Additional file 3) [53] was mapped to 52.5 cM in the NT population, located outside of the QTL3 region. A second QTL for RL-Control was observed on chromosome 7HS (QTL4) with Nure carrying the favourable allele and *Contig_41777* as peak marker. This region is 10 cM away from the main QTL controlling RL under salt stress, QTL5, mapped at 17.2–17.8 cM on the same chromosome, 7HS (Table 2), as detailed in the next section.

To evaluate potential effects of diverse seed sources on the seedling response to salt treatment, 20 DH lines with contrasting RL behaviours were propagated in Reggio Emilia during season 2012–2013, then the DH lines from the two seed sources were subjected to the same seedling experiment. The comparison of RL and SL response curves in tolerant vs. susceptible lines displayed significant differences for RL under 80, 160 and 240 mM NaCl in both seed sources (Additional file 5). Two-way ANOVA showed no significant environmental effect for RL under 80 and 160 mM, and a minor effect (11%) for RL-240 mM (Additional file 6), suggesting that the phenotypic variation for root growth under salt stress is not strongly affected by seed propagation site or year.

### Characterization of the major root length QTL on chromosome 7HS

**Fig. 4:**
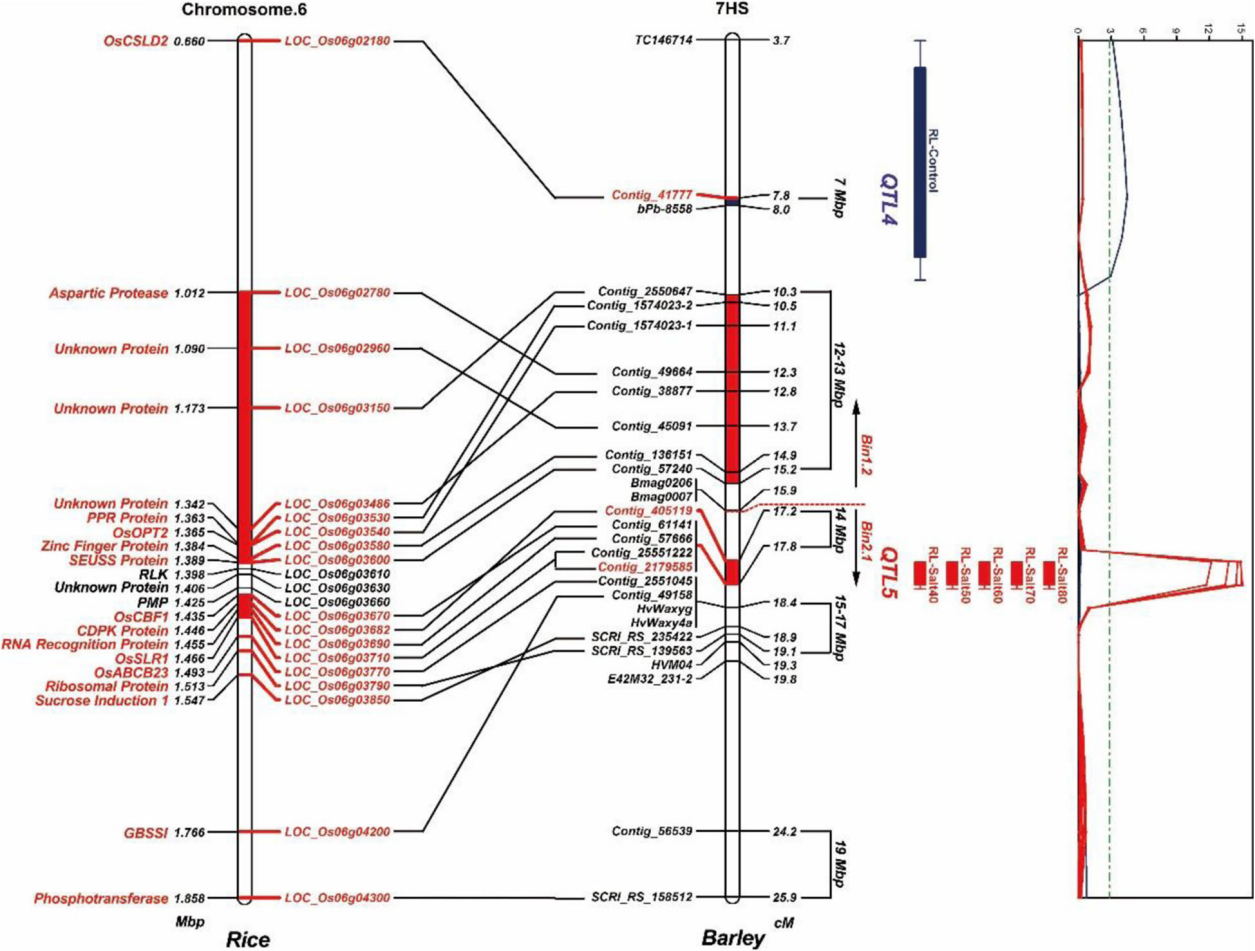
Genomic region of the major QTL detected on chromosome 7HS for RL and its synteny to rice chromosome 6. The LOD profiles from CIM analyses are shown in the *far right panel*. The physical positions of the barley Contig markers are reported according to the recently released sequence of Morex (http://webblast.ipk-gatersleben.de/barley_ibsc/). No sequence polymorphisms between Nure and Tremois were observed for rice genes in *black color*. Barley QTL peak markers are indicated in *red*

To increase marker density around the main QTL region detected on chromosome 7HS for RL under salt stress, the previously published NT molecular map (Francia et al., 2011 [44]) was implemented with 23 new molecular markers based on relationships with the syntenic genomic region of rice chromosome 6S (Fig. 4). The physical positions of these markers in the recently released Morex barley genome [54] and their syntenic rice gene annotations are shown in Additional file 7. No sequence polymorphisms between Nure and Tremois were observed for the homologous barley contigs of three rice genes (*LOC_Os06g03610*, *LOC_Os06g03630* and *LOC_Os06g03660,* from 1.398 Mbp to 1.425 Mbp) and for this reason, a gap is presented on the chromosome 7HS genetic map, between 15.2 cM and 17.2 cM (Fig. 4). Only the *SSR* markers *Bmag0206* and *Bmag0007* mapped to this region, which also served to separate Bin1.2 and Bin2.1 according to the microsatellite consensus map [48]. Higher recombination frequency was observed in Bin1.2 than in Bin2.1, where *Contig_61141*, *Contig_57666*, *Contig_25551222* and *Contig_2179585* in Bin2.1 were mapped to identical positions.

As already noted, a highly significant QTL region was mapped for five RL-REPs (QTL5). *Contig_2179585* was the peak marker for RL-*Salt*_*80*_, which gave the highest LOD value (15.05) and explained 45.3% of the phenotypic variation (Table 2), with the favourable alleles contributed by parent Nure. RL-*Salt*_*70*_ also targeted the same peak marker, while the close *Contig_405119* was mapped as peak marker by the other three traits, with LOD scores from 12.19 to 14.58. Based on the barley Bin map, the syntenic region of the rice genome could be split accordingly: a first segment located on chromosome 6 between 1.012 and 1.389 Mb (*LOC_Os06g02780- LOC_Os06g03600*, 377 kb) is syntenic to barley Bin1.2, while a second region between 1.435 and 1.466 Mb (*LOC_Os06g03670-LOC_Os06g03710*, 31 kb) coincides with Bin2.1. According to the genetic position of QTL5 in Bin2.1, the second rice genomic region may harbour interesting candidate genes, including *LOC_Os06g03670* (dehydration-responsive element-binding protein), *LOC_Os06g03682* (calcium-dependent protein kinase), *LOC_Os06g03690* (RNA recognition motif-containing protein) and *LOC_Os06g03710* (DELLA protein).

### Salt-induced morphological responses in barley seedlings

**Fig. 5:**
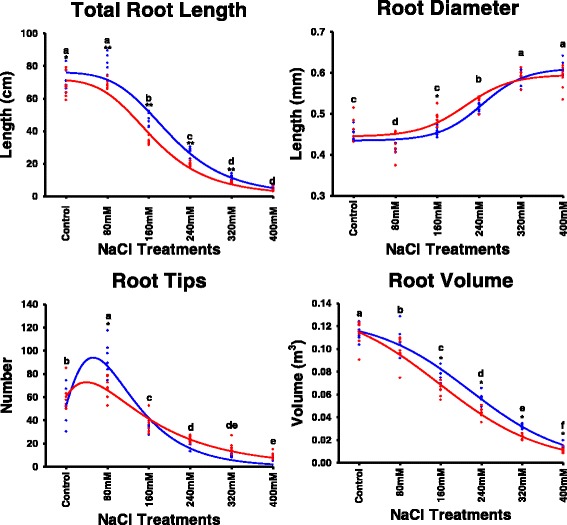
Dynamic response of root parameters in contrasting NT DH lines. Each point represents the observed value after 7 days of seedling growth for six tolerant and six susceptible selected lines. The dynamic curves of total root length and root tips were simulated by Hill 3 parameters and Peak LogNormal model 3 parameters, respectively; for root diameter and root volume, the four parameters and three Sigmoid parameters are adopted, respectively. All fitting coefficients are higher than 0.98. *Blue* and *red colors* refer to salt-tolerant and susceptible groups, respectively. *^,^**Significant differences between tolerant and susceptible groups at *p* < 0.05 and *p* < 0.001, respectively (*t* test). *Letters* represent significant differences between different salt treatments at *p* < 0.05 (Tukey HSD)

Six tolerant and six susceptible DH genotypes were selected based on their contrasting allelic state at molecular markers in the QTL5 region (Additional file 8), and further analysed for TRL, RD, RV and RT to characterize their SIMRs (Fig. 5). TRL was not significantly different between control and 80 mM NaCl treatments. Notably, RT at 80 mM NaCl was significantly higher than the control. In contrast, RV showed a gradual decrease with increasing salt concentration, whereas RD was clearly enhanced by salt concentrations over 240 mM. These results indicate a dynamic pattern of rhizogenesis characterized by shorter and thicker roots in response to increasing salt stress. The phenotypic response under low salinity (80 mM NaCl) suggests the existence of different mechanisms of salt-induced morphological adaptation under this condition.

**Fig. 6:**
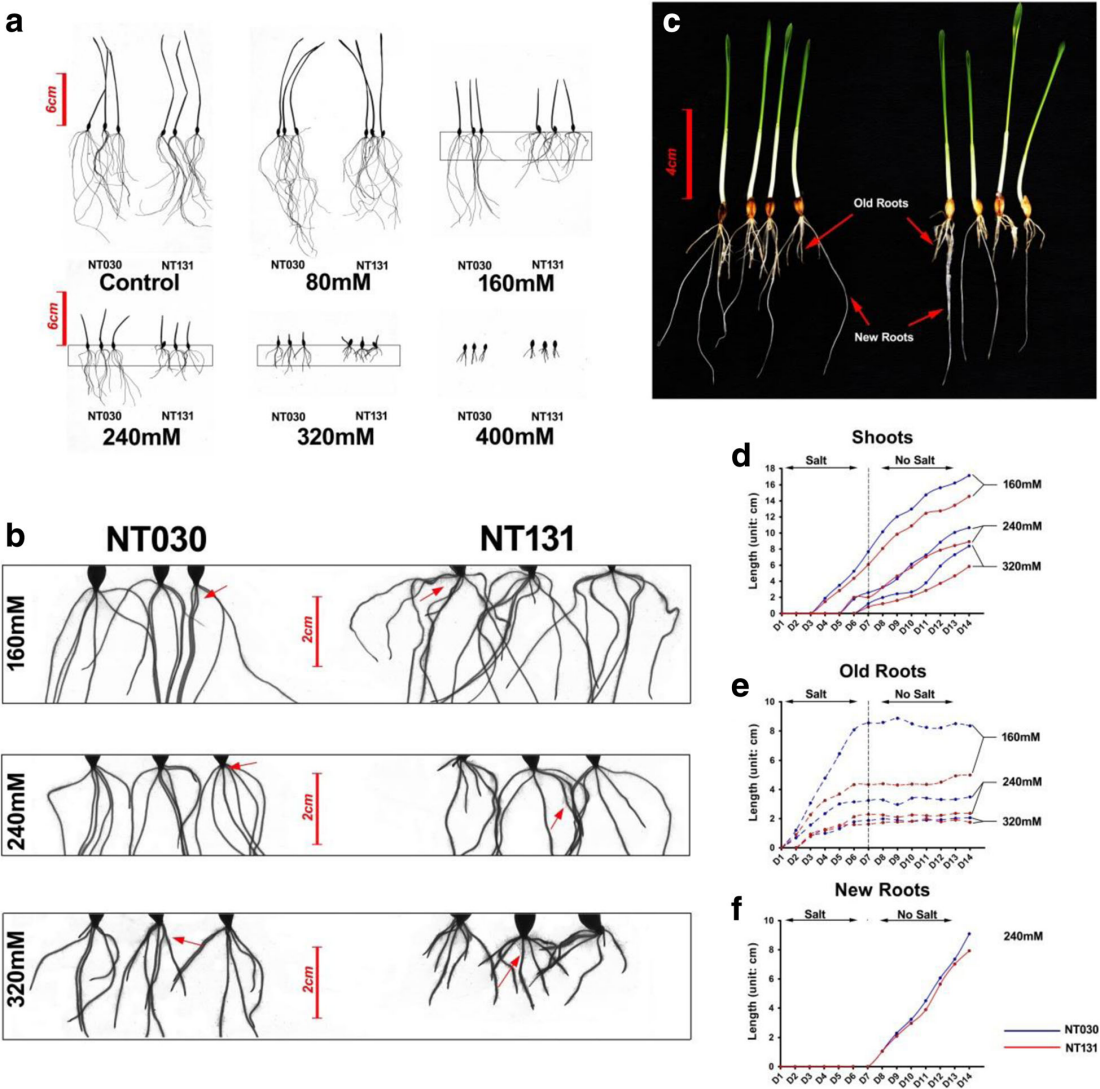
Salt-induced morphological responses of two contrasting genotypes under different NaCl treatments. **a** NT030 (tolerant) and NT131 (susceptible) DH genotypes were germinated at the indicated NaCl treatments; all images were scanned after 7 days. **b** Root hairs of two genotypes from 160, 240 and 320 mM NaCl treatment were compared at the points indicated by the red arrows. **c** Seedlings germinated for 7 days in 240 mM NaCl were transferred to no-salt conditions for an additional 7 days; old (yellow) and new (white) roots are indicated. **d–f** Root and shoot lengths under 160 mM, 240 mM and 320 mM NaCl treatment were observed for 14 days (7 days of salt-stress conditions plus 7 days of recovery without salt). Blue and red lines refer to NT030 and NT131, respectively. All values are averages of three replications. Scale bars in **a**, **b**, **c** = 6, 2 and 4 cm, respectively

Figure 6 shows the seedling behaviours of two extreme genotypes (NT030 and NT131) after 7 days of salt-stressed seedling growth and an additional 7 days of “recovery” (only purified water). Consistent with the results reported in Fig. 2 for the whole population, there were no clear differences between the two genotypes at either 0 or 80 mM NaCl (Fig. 6a) during the first 7 days, whereas RL of the susceptible line NT131 was clearly shorter than that of the tolerant NT030 line under salt-stress conditions of 160, 240 and 320 mM NaCl. No similar patterns were observed for SL during the 7 treatment days (Fig. 6a). The dynamic growth curves of SL and RL (Fig. 6d and e) confirmed only minor differences in SL between the two lines, but much larger variations in RL starting from day 4. Along with a decrease in RL under 160, 240 and 320 mM NaCl, both NT030 and NT131 showed an increase in root hair (RH) density (Fig. 6b, red arrows). Higher RH density was observed in NT131 compared to NT030 under all three salt treatments, while there was no clear difference between the two lines at 0 mM (data not shown), suggesting that the shorter roots of the susceptible lines might be associated with increased RH density. Notably, new roots emerged (Fig. 6c) and grew rapidly in both lines during the recovery phase (Fig. 6f), whereas the existing roots did not elongate further (Fig. 6e).

## Discussion

The genetic distances, different growth habits and different seed usages of Nure (winter feeding barley) and Tremois (spring malting barley) make the corresponding segregating population an ideal tool for the genetic dissection of many agronomic traits, e.g., frost tolerance, malting quality, yield and yield components [41–45]. In addition to the successes in these traits, a preliminary experiment also highlighted the differing seedling growth capacities of Nure and Tremois under salt stress, presenting an aspect for the genetic analysis of loci controlling salt tolerance at seedling stage. With the aim of improving mapping accuracy, a novel and comprehensive phenotyping protocol was developed to describe the dynamic seedling response to salt stress, and the key parameters of the seedling curves were employed for QTL analysis.

### The dynamic response of barley seedlings to salt stress

When seeds are germinated in petri dishes or open plastic boxes, their growth can be impaired by space limitations, making RL measurements unreliable. On the other hand, the vertical germination system does not limit the elongation of either roots or shoots and allows direct observations of seedling growth. The *Arabidopsis* Germinator platform [39] can largely improve the work efficiency, with high-throughput automatic evaluation of germinating seeds. Here, we combined the vertical germination system with photographic acquisition of final seedling length and ImageJ software for trait measurements to shorten the processing time and enhance data accuracy.

In previous studies, only one or a few salt concentrations were used to test the effects of NaCl on barley seedlings. For example, 1.5% (256 mM), 2% (341 mM) and 2.5% (427 mM) NaCl solutions were used to screen the OWB population [23]; 250 mM and 300 mM NaCl solutions were used for the SM [31] and other recombinant inbred line populations [55]. Recently, the OWB and L94 × Vada populations were examined with concentrations of 0, 200 and 300 mM NaCl [34]. Thus, salt concentrations between 200 and 300 mM NaCl are most frequently selected for stress treatments, without first identifying the most suitable concentration leading to the best phenotypic variation, for more efficient QTL mapping. For instance, the largest data range was observed with the 160 mM NaCl treatment in the present work (Fig. 2), a concentration that was not explored in previous studies. Similarly, low salt (e.g., 80 mM) concentrations are not usually considered. In our study, RL-Control and RL-80 mM did not differ significantly (Fig. 2), but a unique root SIMR of barley seedlings in the presence of 80 mM NaCl was observed (Fig. 5).

No published works have ever depicted the dynamic curves for barley seedling length (RL and SL) plotted against different salt concentrations, a simulation that integrates the phenotypic values from each individual salt treatment (Fig. 1). The concept of dynamic seedling growth curves is derived from studies on seed dormancy [56]. Unlike *G*_*max*_, *L*_*max*_ is genotype-dependent, leading to different intercepts for the simulated curves (Fig. 1). Similarly, REPs extracted from the Hill equation model are very useful for screening germplasm collections with large genetic diversity; for instance, small roots from particular genotypes might not be dramatically reduced by salt conditions, resulting in “flatter” curves, such as the curve of NT123 in Fig. 1. In the current work, the REPs gave the highest LOD values and *R* ^2^ scores in the NT mapping population, confirming their power in accurately describing seedling growth under salt stress.

### QTLs and candidate genes for barley seedling growth under salt stress

Two QTLs were mapped in the present study at different genetic positions on chromosome 7HS (QTL4 and QTL5). The peak marker of QTL4 for RL-Control was *Contig_41777* targeting a cellulose synthase-like protein-encoding gene (*HvCSLD2*), according to the syntenic rice gene annotation [57]. A positive role for *AtCSLD2* on RH growth has been shown in *Arabidopsis* [58], while in barley, *HvCSLD2* mediated penetration resistance of leaf cell walls to powdery mildew, but no effects on RH formation were observed in transgenic barley plants expressing *HvCSLD2* RNAi [59]. Hence, its role in seedling root development has yet to be confirmed. A major QTL for seminal root angle has been recently mapped in a barley advanced backcross population close to the *DArT* marker *bPb-8558* [60], which was also located at QTL4 in our study (Fig. 4).

Barley chromosome 7HS has been previously associated with several malting quality traits [61, 62]. Since malting is a specialized germination process, a malting quality QTL might also be involved in initial seedling growth in presence of salinity. For example, the QTL for diastatic power was mapped to the *Bmag007* region with a high LOD value [63]. A QTL explaining 61.8% of the phenotypic variations for β-glucan was also detected on chromosome 7HS, with *granule-bound starch synthase* (*GBSSI*) as a promising candidate gene [64]. Both *GBSSI* and its closely linked *SSR* marker *HvWaxyg* were mapped in the NT population only 0.62 cM away from QTL5 on Bin2.1 (Fig. 4). Nevertheless, the NT population has been previously exploited for studying the genetic bases of malting quality, and no QTLs were detected on chromosome 7H [45]. A correlation analysis between malting quality data [45] and RL data under salt stress did not show any significant results (data not shown). Hence, we hypothesize that the loci controlling malting traits previously identified on chromosome 7HS do not segregate in the NT population and therefore, the major QTL detected in this study for RL under salt stress is not related to malting quality.

The peroxidase (POD)-mediated ROS system [65] might regulate α-amylase activity in the seed endosperm through hormone signalling [66]; suppression of POD activity under salt stress could disrupt the balance of the ROS system in roots, thus retarding seedling growth and resulting in poor germination. In barley, a proteomic analysis has recently proven that POD is induced in 6-day-old seedlings under salt stress [67]. Moreover, a *p*QTL for the accumulation of POD precursors has been mapped on chromosome 7HS linked to the *SSR* marker *Bmag007* [68]. The *CAPS* marker developed from the *POD BP1* gene (*Contig_56539*) was mapped on the same chromosome, 7HS, at 24.2 cM, which is 6.41 cM away from QTL5 (Fig. 4). This suggests that the POD precursor gene *BP1* is not a candidate for the major QTL identified in this study.

QTLs and genes involved in salt tolerance in wheat, rice and barley at different developmental stages have been summarized [69]. Several genes involved in ion homeostasis play key roles in salt tolerance at the vegetative stage, e.g. *HKT* (*high-affinity K* ^*+*^ *transporter*), *NHX* (*Na* ^*+*^ */H* ^*+*^ *exchanger*), *SOS* (*salt overly sensitive*) and *Nax* (*sodium exclusion*). Of these, only *HvNax3* is located on barley chromosome 7HS. *HvNax3* can reduce Na^+^ concentration in shoots, with the positive allele derived from wild barley [22]. Its candidate gene, *Hordeum vacuolar H* ^*+*^ *-pyrophosphatase* (*HVP10*) is located on *Contig_2547568*, mapped at a position proximal to the centromere but distant from the QTL5 region detected in the present study.

*C-repeat binding factor* (*CBF*) is a large gene family playing multiple roles in tolerance to low temperature [47], drought and salt stress [70]. Several *CBF* genes have also been mapped in the NT population, clustered on chromosome 5HL [71] where no QTLs for salt tolerance were detected (Fig. 3). However, *HvCBF5* has been mapped on 7HS in the Dicktoo × Morex population [72], and *Contig_61141*, which hosts this gene, was mapped here within the QTL5 region (Fig. 4). The *HvCBF5* sequences in Nure and Tremois differ by two *SNPs* within the CDS region. However, it should be noted that when this mapping population was screened for seedling growth under PEG_6000_-mediated osmotic stress, no QTLs were detected on chromosome 7HS (data not shown), which makes *HvCBF5* a weak candidate gene for QTL5, based on its known dehydration-responsive function.

On rice chromosome 6S, the region from 1.435 to 1.466 Mb syntenic to QTL5 contains another two potential candidate genes, *CDPK* (calcium-dependent protein kinase, *LOC_Os06g03682*) and *OsSLR1* (*Slender Rice 1*, *LOC_Os06g03710)*. The involvement of *CDPK*s in the response to abiotic stress has been analysed in detail [73], in particular for their important roles in signalling pathways and ABA-dependent regulation of seed germination. Moreover, *HvCDPK1* mediates the gibberellin (GA) response of barley aleurone through cell vacuolation and vacuolar acidification and is involved in GA regulation during seed germination [74]. The rice DELLA protein OsSLR1 has also been indicated as a direct negative modulator of GA as well as a regulator of α-amylase activity during seed germination [75, 76]. Five *SNPs* have been detected between Nure and Tremois within the CDS region of *MLOC_56062*, a CDPK-like protein-encoding gene on *Contig_405119*, whereas 49 *SNPs* have been observed in *HvSLR1*, an ortholog of *OsSLR1*. To date, known examples of DELLA proteins are SLN1 in barley [77], SLR1 in rice [78], RGA/GAI/RGL in *Arabidopsis* [79], and Rht-B1/Rht-D1 in wheat [80]. All of these proteins share the GRAS domain interacting with the GID1 receptor and GA, leading to a GA–GID1–DELLA complex [81] and resulting in low bioactive GA levels. In barley, *HvGAMYB* repression by SLN1 is associated with lower α-amylase activity [77]. The degradation of SLN1 by a SCF E3 ubiquitin ligase is also crucial for GA regulation [82]. Some *sln*1 mutants show higher GA levels, leading to over-growing plants [83]. The *SNP* variations observed between Nure and Tremois might lead to significant alterations in protein structure, including the motif which triggers DELLA’s degradation, resulting in different degradation efficiencies between parents under salt stress. Whether *HvSLR1* is the candidate gene for QTL5 will be the focus of future studies.

### Morphological response of barley seedlings to salt stress

Pasternak [84] pointed out that plant responses to salt stress not only result in growth cessation but also in growth resumption, albeit possibly at a reduced rate [16], and our results support this finding. Root elongation ceased or was retarded by salt stress, resulting in reductions in TRL, RT numbers and RV, but the increase in RD and RH density might represent a re-growth response which has never been studied in barley. The SIMR of *Arabidopsis* roots to Cu highlight increased RH density in axillary roots, and the altered auxin profile suggested that the roots are in the process of growth re-orientation rather than cessation [84]. Decreased root elongation and increased thickness have also been observed in the SIMR of *Miscanthus sinensis* to Cd stress [85]. The RTs of wheat seedlings are thickened by exposure to increasing concentrations (from 5 to 15%) of PEG_8000_ [86], and similar results have also been observed in *Arabidopsis* for RH density. The similarities in RH response between drought stress and our salt treatment suggest that it might be evoked by osmotic components.

As shown in Fig. 5 and Additional file 5, there were no significant differences between TRL and RL under 0 and 80 mM NaCl, whereas the number of RTs increased significantly, and the opposite behaviour was observed for RD. This SIMR of roots under 80 mM NaCl differs from the pattern of modifications detected at higher salt concentrations. Similar results were also observed in 10-day-old seedlings of the maize accession Zeo 6 under different salt concentrations, with no clear RL decrease below 40 mM NaCl [87]. However, the increased number of RTs observed at 80 mM in our study might be due to a perturbation in homeostasis from Na^+^ or Cl^−^ ions rather than the osmotic stress, since the latter appears to inhibit lateral root formation but not increase RT numbers [88]. Notably, a SIMR study in *Arabidopsis* roots showed that an intermediate level of salt stress can increase the number of lateral roots, together with a drastic reduction in primary and lateral root elongation [89], indicating the ionomic effects of salt-stimulated formation of RTs. Different SIMRs at low and high salt concentrations suggest independent genetic control.

Six tolerant and six susceptible selected lines (based on haplotypes at QTL5) also exhibited significant differences in TRL under control, 80, 160, 240 and 320 mM NaCl conditions, in RT number at 80 mM NaCl, and in RV at 160, 240, 320 and 400 mM NaCl (Fig. 5). These results also suggest that QTL5 might significantly impact TRL, RT number and RV under those conditions.

During the recovery phase after salt stress, the barley seedling produced new roots instead of further elongating the old ones (Fig. 6c). This particular phenotype was very clear in plants germinated in 160, 240 and 320 mM NaCl, whereas at 80 mM NaCl, new roots grew and old roots elongated simultaneously. Thus root growth that ceased under high salinity stress could not be restored by removal of the stress, similar to the response in barley cultivar Noga after rehydration [90]. However, no significant variations during recovery were observed between the two investigated DH lines, suggesting that the QTL for salt tolerance has no effect on re-growth when the stress is removed.

## Conclusions

We present a novel comprehensive method for studying the responses of barley seedlings to different salt concentrations. Its application led to the identification of a major new QTL on chromosome 7HS in the NT mapping population. Furthermore, genotypes with contrasting behaviours were profiled for root morphological traits, providing useful insights into barley seedling growth under saline conditions. The major QTL previously detected on chromosome 5H in 10-day-old seedlings grown under salt stress [23] is very close to the QTL controlling seed dormancy, *HvQsd1* (*QTL seed dormancy 1*), which encodes an *alanine aminotransferase* gene [33]. Together with the candidate *HvSLR1* gene for the major QTL in this study, both genes seem irrelevant for ion homeostasis, according to the morphological responses of seedlings to moderate salt stress. Hence, enzymatic or hormonal regulation by functional proteins might play a key role in the morphological responses to salt stress at initial seedling stages.

## Additional files

Additional file 1: A novel experimental system for vertical seed germination. (A) A plate for vertical seed germination. (B) Different boxes are used for the different salt treatments, with 22 plates in each box (C, D), including two blank plates in the first and last positions to reduce unbalanced evaporation rates within boxes. (E) Seeds are sown inside a paper hood. All material used in this system was non-metallic. (DOCX 232 kb)

Additional file 2: Photographs of 7-day-old seedlings after germination in 160 mM NaCl. Left and right positions represent NT071 and NT084, respectively. Red lines indicate measurements of root and shoot length by the “Segmented Line” function of ImageJ. The scale bar of 4 cm is labelled within photo. (DOCX 339 kb)

Additional file 3: Details of 25 molecular markers mapped in the present study on the NT genetic map. (DOCX 25 kb)

Additional file 4: Descriptive statistics on REPs in parental line and whole DH population. (DOCX 17 kb)

Additional file 5: Ten tolerant and ten susceptible DH lines with two different seed sources were evaluated for their dynamic response of RL and SL under different NaCl treatments. Results A obtained with seeds harvested in 2013, and results B obtained with seeds harvested in 2007. Each point represents the length data after 7 days of germination for the 20 selected lines. Tolerant and susceptible groups are distinguished by blue and red colors respectively. Significant differences (*p* < 0.05 and *p* < 0.001) between tolerant and susceptible groups are indicated by “*” and “**”, respectively. Letters on the graph denote statistically significant differences between NaCl treatments (Tukey HSD, *p < 0.05*). (DOCX 45 kb)

Additional file 6: Two-way ANOVA on RL of 20 selected DH lines from two environments. (DOCX 14 kb)

Additional file 7: List of 23 gene-based molecular markers and their syntenic gene annotations in rice genome. (DOCX 16 kb)

Additional file 8: Allelic state of selected tolerant and susceptible NT lines for molecular markers linked to the major QTL on chromosome 7HS. “N” and “T” represent the alleles from the parent Nure and Tremois, respectively. (DOCX 15 kb)

## Abbreviations

*CAPS*: Cleaved amplified polymorphic sequence
*CBF*: C-repeat binding factor
*CDPK*: Calcium-dependent protein kinase
CIM: Composite interval mapping
*dCAPS*: Derived-cleaved amplified polymorphic sequence
DH: Double-haploid
*G*_*max*_: Maximum germination percentage
*H*^*2*^: Heritability
IM: Interval mapping
*L*_*max*_: Maximum seedling length
LOD: Logarithm of odd
NT: Nure × Tremois
OWB: Oregon Wolfe barley
POD: Peroxidase
QTL: quantitative trait loci
RD: Root diameter
REP: Response parameter
RH: Root hair
RL: Seminal root length
ROS: Reactive oxygen species
RT: Root tips
RV: Root volume
SIMR: Stress-induced morphological responses
SL: Shoot length
*SLR1*: *Slender Rice 1*
SM: Steptoe × Morex
*SNP*: Single nucleotide polymorphism
*SSR*: Simple sequence repeat
*STS*: Sequence-tagged sites
TRL: Total root length
