# Supplementary material for: A major QTL on chromosome 7HS controls the response of barley seedling to salt stress in the Nure × Tremois population
Source: BMC Genet. 2017 Aug 22;18:79. doi: 10.1186/s12863-017-0545-z (PMC5568257; doi:10.1186/s12863-017-0545-z)
Supplement: Supplementary file 1 — A novel experimental system for vertical seed germination. (A) A plate for vertical seed germination. (B) Different boxes are used for the different salt treatments, with 22 plates in each box (C, D), including two blank plates in the first and last positions to reduce unbalanced evaporation rates within boxes. (E) Seeds are sown inside a paper hood. All material used in this system was non-metallic. (DOCX 232 kb) [file 12863_2017_545_MOESM1_ESM.docx]

**
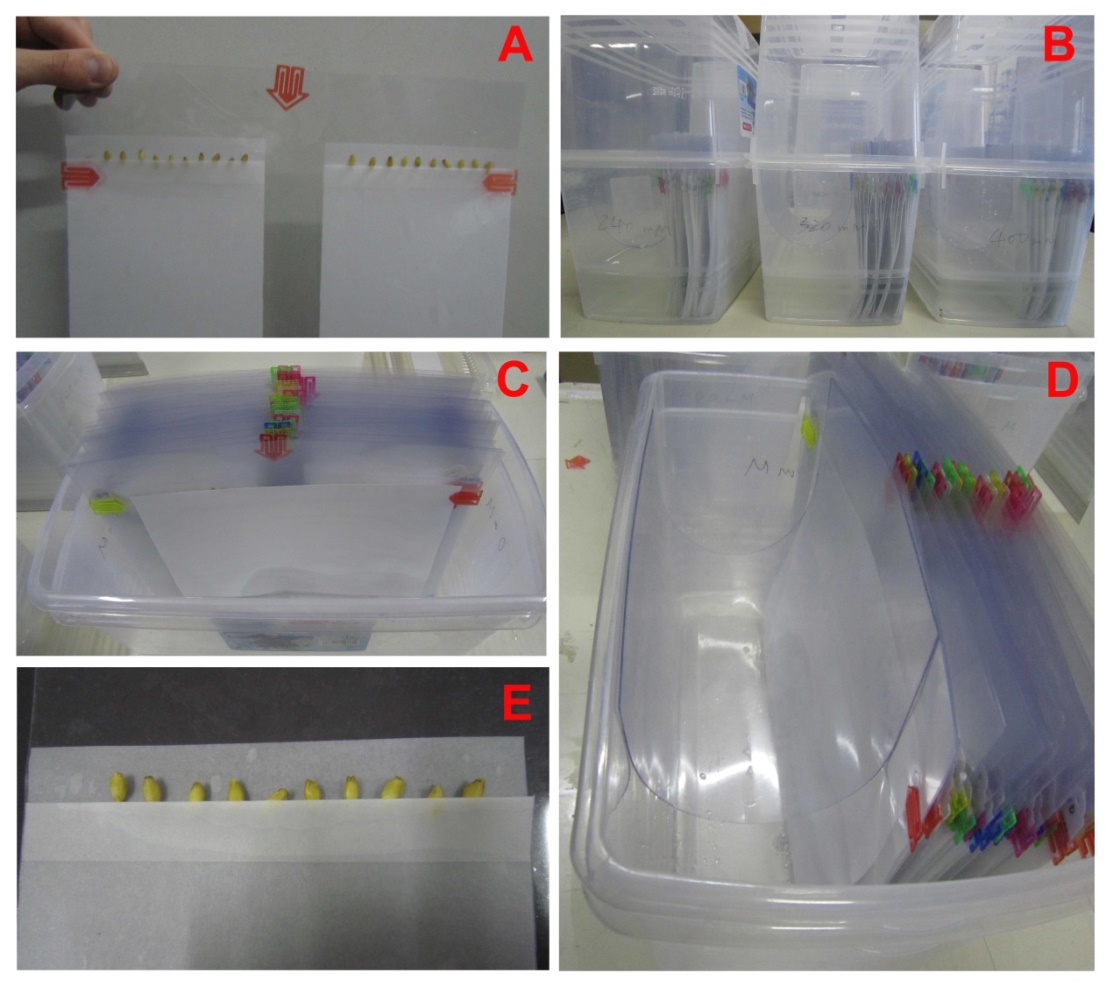
**

**Additional file 1. A novel experimental system for vertical seed germination. (A)** A plate for vertical seed germination. **(B)** Different boxes are used for the different salt treatments, with 22 plates in each box **(C, D)**, including two blank plates in the first and last positions to reduce unbalanced evaporation rates within boxes. **(E)** Seeds are sown inside a paper hood. All material used in this system was non-metallic.
