# Supplementary material for: A major QTL on chromosome 7HS controls the response of barley seedling to salt stress in the Nure × Tremois population
Source: BMC Genet. 2017 Aug 22;18:79. doi: 10.1186/s12863-017-0545-z (PMC5568257; doi:10.1186/s12863-017-0545-z)
Supplement: Supplementary file 2 — Photographs of 7-day-old seedlings after germination in 160 mM NaCl. Left and right positions represent NT071 and NT084, respectively. Red lines indicate measurements of root and shoot length by the “Segmented Line” function of ImageJ. The scale bar of 4 cm is labelled within photo. (DOCX 339 kb) [file 12863_2017_545_MOESM2_ESM.docx]

**
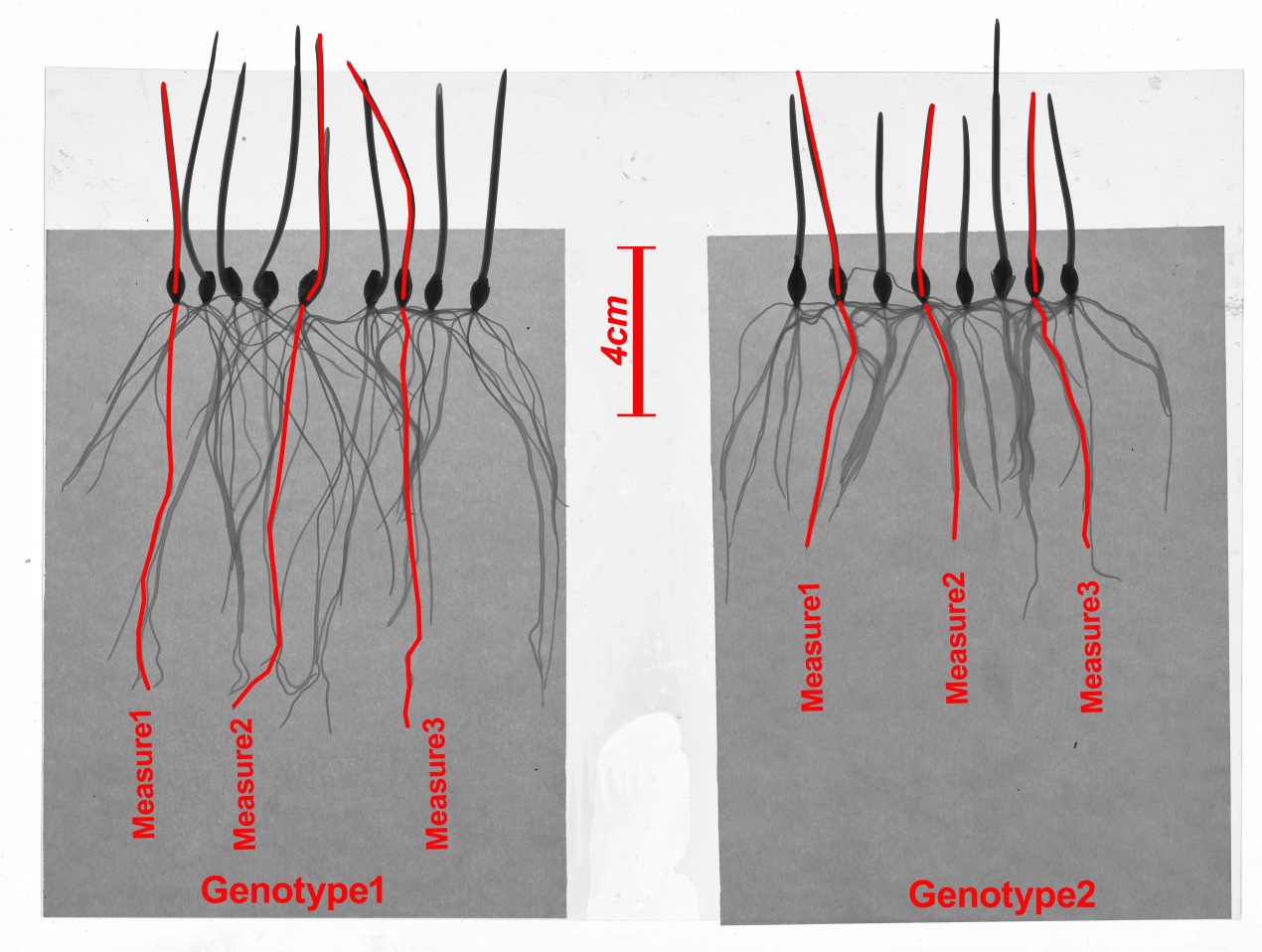
**

**Additional file 2. Photographs of 7-day-old seedlings after germination in 160 mM NaCl.** Left and right positions represent NT071 and NT084, respectively. Red lines indicate measurements of root and shoot length by the “Segmented Line” function of ImageJ. The scale bar of 4 cm is labeled within photo.
