# Supplementary material for: A major QTL on chromosome 7HS controls the response of barley seedling to salt stress in the Nure × Tremois population
Source: BMC Genet. 2017 Aug 22;18:79. doi: 10.1186/s12863-017-0545-z (PMC5568257; doi:10.1186/s12863-017-0545-z)
Supplement: Supplementary file 3 — Details of 25 molecular markers mapped in the present study on the NT genetic map. (DOCX 25 kb) [file 12863_2017_545_MOESM3_ESM.docx]

**Additional file 3. Details of 25 molecular markers mapped in the present study on the NT genetic map.**

| **Maker Name** | **Position in NT** | **Type** | **Primers** | **PCR Procedure** |
| --- | --- | --- | --- | --- |
| *BOPA2_12_30080* | 5H, 52.5 cM | *STS* | F: cataagccccaccttcctctc  R: accagagagtaacaccgactt | ST^a^ |
| *GBS0318* | 5H, Not Mapped | *STS* | F: gctgagaagattgtccagat  R: agcttttaaagttcatgccg | ST |
| *^*^Contig_41777* | 7H, 7.8 cM | *CAPS* | F: gtctatgtgtcccgagaaaa  R: tcctctgtgactgaaccata | TD^b^, *PstI* ^c^ |
| *^*^Contig_2550647* | 7H, 10.3 cM | *STS* | F: gcgtttcaatcctgtctagt  R: aaacagggcctaattccttt | ST |
| *^*^Contig_1574023-2* | 7H, 10.5 M | *CAPS* | F: taccccatgatgtactctgt  R: tcccagttccatttcgatac | TD, *HpaII*^c^ |
| *^*^Contig_1574023-1* | 7H, 11.1 cM | *CAPS* | F: aaggtactcgacgaaatgcc  R: cgtaaatactgcagcccctt | TD, *BstEII*^c^ |
| *^*^Contig_49664* | 7H, 12.3 cM | *CAPS* | F: ccgtgtcagctacatccaaa  R: tatctggaggcgtcgattct | TD, *PstI*^c^ |
| *^*^Contig_38877* | 7H, 12.8 cM | *STS* | F: taatcagccaacagacaaca  R: tgtgacttattccagagcag | LT^d^ |
| *^*^Contig_45091* | 7H, 13.7 cM | *CAPS* | F: cagcacctttccaagcacta  R: ttgtcgtctacgagcatgtg | TD, *BstEII*^c^ |
| *^*^Contig_136151* | 7H, 14.9 cM | *dCAPS* | F: gcattagctagtctttccga  dR: tcttattatcagaggatttac***gg***ttac | LT, *BstEII*^c^ |
| *^*^Contig_57240* | 7H, 15.2 cM | *dCAPS* | F: gctgtgtcctgtctagttag  dR: tgaattacaaactagcacatagataca***c***tca | TD, *DdeI*^c^ |
| *Bmag0206* | 7H, 15.9 cM | *SSR* | F: ttttcccctattatagtgacg  R: tagaactgggtatttccttga | TD |
| *^*^Contig_405119* | 7H, 17.2 cM | *CAPS* | F: gctcggcgatctactataag  R: atcctccttctctgcgttta | LT, *HpaII*^c^ |
| *^*^Contig_61141* | 7H, 17.8 cM | *dCAPS* | F: ggctggcatggatctttctt  dR: gatgagcggcggcggcgactggcaga***ag***atc | TD, *BglII*^c^ |
| *^*^Contig_57666* | 7H, 17.8 cM | *CAPS* | F: taatgcagcatacaagagca  R: tttagtggtcattctgaggc | LT, *CfoI*^c^ |
| *^*^Contig_2555122* | 7H, 17.8 cM | *STS* | F: caacggtaggaaacggacg  R: ttggactctgcagaaatgct | LT |
| *^*^Contig_2179585* | 7H, 17.8 cM | *CAPS* | F: gtggtatggttccatcaatc  R: ccttgcttgatgtcgaagt | TD, *NarII*^c^ |
| *^*^Contig_2551045* | 7H, 18.4 cM | *CAPS* | F: tggatcttctacttgcgatg  R: agaataatcccattcccgtg | LT, *RsaI*^c^ |
| *^*^Contig_49158* | 7H, 18.4 cM | *CAPS* | F: ggtagttgctcttgaggtag  R: ggtatccgccatatgaatga | TD, *AluI*^c^ |
| *HvWaxyg* | 7H, 18.4 cM | *SSR* | F: tccaatggcatctacaggacggccaa  R:gcaggttgagctgcgcaaagtcgtcg | ST |
| *HvWaxy4a* | 7H, 18.4 cM | *SSR* | F: agtatcgcagacgctcac  R: gttatgtactcgctcgctc | ST |
| *SCRI_RS_235422* | 7H, 18.9 cM | *CAPS* | F: ttggaaggttgttgcctttt  R: aggtacagaccagctaaaga | TD, *TaqI*^c^ |
| *SCRI_RS_139563* | 7H, 19.1 cM | *dCAPS* | dF: agcagccgtagcagcattagca***ga***tat  R: atgcatatcacattgtgcaaac | LT, *EcoRV*^c^ |
| *^*^Contig_56539* | 7H, 24.2 cM | *CAPS* | F: cataagccccaccttcctctc  R: accagagagtaacaccgactt | TD, *XhoI*^c^ |
| *SCRI_RS_158512* | 7H, 25.9 cM | *CAPS* | F: gccacttccactgcttat  R: gagttgagcggaaccttg | TD, *MboI*^c^ |

^a^ Standard procedure: 94 ℃ 5 min; 35 cycles of 94 ℃ 30 s, 56 ℃ 30 s and 72 ℃ 30 s; 72 ℃ 5 min.

^b^ Touch-Down procedure: 94 ℃ 5 min; touch-down from 60 to 50 ℃ for every 1 ℃, 2 cycles at each degree, each cycle set as 94 ℃ 1 min, 60 to 50 ℃ 1 min and 72 ℃ 1 min; 30 cycles of 94 ℃ 1 min, 50 ℃ 1 min and 72 ℃ 1 min; 72 ℃ 5 min.

^c^ Restriction endonuclease.

^d^ Long-Term procedure: 94 ℃ 5 min; 5 cycles of 94 ℃ 1 min, 58 ℃ 2 min and 72 ℃ 1 min 30 s; 30 cycles of 94 ℃ 1 min, 56 ℃ 2 min and 72 ℃ 1 min 30 s; 72 ℃ 5min.

^*^All contigs used template of the Morex IBSC 2012 gene space assembly for primer design.
