# Supplementary material for: A major QTL on chromosome 7HS controls the response of barley seedling to salt stress in the Nure × Tremois population
Source: BMC Genet. 2017 Aug 22;18:79. doi: 10.1186/s12863-017-0545-z (PMC5568257; doi:10.1186/s12863-017-0545-z)
Supplement: Supplementary file 4 — Descriptive statistics on REPs in parental line and whole DH population. (DOCX 17 kb) [file 12863_2017_545_MOESM4_ESM.docx]

**Additional file 4. Descriptive statistics on REPs in parental line and whole DH population.**

| REPs | Nure | Tremois | Mean ± S. D | Range | *H^2^* |
| --- | --- | --- | --- | --- | --- |
| RL-*L_max_* (cm) | 13.13 | 13.27 | 13.26 ± 0.61 | 12.01-14.78 | 0.64 |
| RL-*Salt_80_* (mM) | 146.16 | 123.24 | 136.55 ± 18.68 | 94.92-179.32 | 0.71 |
| RL-*Salt_70_* (mM) | 172.97 | 149.08 | 162.65 ± 18.43 | 120.29-203.33 | 0.71 |
| RL-*Salt_60_* (mM) | 197.35 | 172.94 | 186.61 ± 18.03 | 144.81-224.68 | 0.72 |
| RL-*Salt_50_* (mM) | 221.83 | 197.21 | 210.82 ± 17.57 | 169.39-247.31 | 0.72 |
| RL-*Salt_40_* (mM) | 248.51 | 223.90 | 237.45 ± 17.16 | 196.82-273.22 | 0.72 |
| RL-*Salt_30_* (mM) | 280.27 | 256.02 | 269.36 ± 17.00 | 224.52-303.89 | 0.71 |
| RL-*Salt_20_* (mM) | 323.23 | 300.05 | 312.99 ± 17.70 | 259.73-346.55 | 0.68 |
| SL-*L_max_* (cm) | 13.02 | 13.26 | 12.50 ± 0.78 | 10.43-14.37 | 0.67 |
| SL-*Salt_80_* (mM) | 93.72 | 102.16 | 101.30 ± 12.37 | 74.95-136.95 | 0.56 |
| SL-*Salt_70_* (mM) | 111.40 | 123.42 | 123.15 ± 12.46 | 96.05-158.54 | 0.58 |
| SL-*Salt_60_* (mM) | 127.30 | 142.79 | 143.20 ±12.59 | 115.92-177.85 | 0.60 |
| SL-*Salt_50_* (mM) | 143.09 | 162.25 | 163.45 ± 12.91 | 136.38-196.98 | 0.63 |
| SL-*Salt_40_* (mM) | 160.11 | 183.45 | 185.63 ± 13.57 | 158.09-220.19 | 0.66 |
| SL-*Salt_30_* (mM) | 180.16 | 208.70 | 212.20 ± 14.88 | 180.16-258.62 | 0.68 |
| SL-*Salt_20_* (mM) | 206.93 | 242.83 | 248.35 ± 17.61 | 206.93-315.22 | 0.70 |
