# Supplementary material for: A major QTL on chromosome 7HS controls the response of barley seedling to salt stress in the Nure × Tremois population
Source: BMC Genet. 2017 Aug 22;18:79. doi: 10.1186/s12863-017-0545-z (PMC5568257; doi:10.1186/s12863-017-0545-z)
Supplement: Supplementary file 5 — Ten tolerant and ten susceptible DH lines with two different seed sources were evaluated for their dynamic response of RL and SL under different NaCl treatments. Results A obtained with seeds harvested in 2013, and results B obtained with seeds harvested in 2007. Each point represents the length data after 7 days of germination for the 20 selected lines. Tolerant and susceptible groups are distinguished by blue and red colors respectively. Significant differences (p < 0.05 and p < 0.001) between tolerant and susceptible groups are indicated by “*” and “**”, respectively. Letters on the graph denote statistically significant differences between NaCl treatments (Tukey HSD, p < 0.05). (DOCX 45 kb) [file 12863_2017_545_MOESM5_ESM.docx]

**Additional file 5. Ten tolerant and ten susceptible DH lines with two different seed sources were evaluated for their dynamic response of RL and SL under different NaCl treatments.** Results A obtained with seeds harvested in 2013, and results B obtained with seeds harvested in 2007. Each point represents the length data after 7 days of germination for the 20 selected lines. Tolerant and susceptible groups are distinguished by blue and red colors respectively. Significant differences (*p*<0.05 and *p*<0.001) between tolerant and susceptible groups are indicated by “*” and “**”, respectively. Letters on the graph denote statistically significant differences between NaCl treatments (Tukey HSD, *p<0.05*).
