# Supplementary material for: A major QTL on chromosome 7HS controls the response of barley seedling to salt stress in the Nure × Tremois population
Source: BMC Genet. 2017 Aug 22;18:79. doi: 10.1186/s12863-017-0545-z (PMC5568257; doi:10.1186/s12863-017-0545-z)
Supplement: Supplementary file 6 — Two-way ANOVA on RL of 20 selected DH lines from two environments. (DOCX 14 kb) [file 12863_2017_545_MOESM6_ESM.docx]

**Additional file 6. Two-way ANOVA on RL of 20 selected DH lines from two environments.**

|  | Environment | | Genotype | | E ×G | | Error | Total |
| --- | --- | --- | --- | --- | --- | --- | --- | --- |
|  | SS | % ^a^ | SS | % ^a^ | SS | % ^a^ | SS | SS |
| DF | 1 | | 19 | | 19 | | 80 | |
| RL-80mM | 3 | 2 | 95** | 60 | 17 | 11 | 45 | 160 |
| RL-160mM | 3 | 1 | 353** | 75 | 39* | 8 | 77 | 473 |
| RL-240mM | 11* | 5 | 148** | 69 | 13 | 6 | 41 | 213 |

^a^ Percentage of SS (sum of squares) was calculated by dividing by total SS.

*^,^**Significant differences at *p* < 0.05 and *p* < 0.001, respectively.
