# Supplementary material for: A major QTL on chromosome 7HS controls the response of barley seedling to salt stress in the Nure × Tremois population
Source: BMC Genet. 2017 Aug 22;18:79. doi: 10.1186/s12863-017-0545-z (PMC5568257; doi:10.1186/s12863-017-0545-z)
Supplement: Supplementary file 7 — List of 23 gene-based molecular markers and their syntenic gene annotations in rice genome. (DOCX 16 kb) [file 12863_2017_545_MOESM7_ESM.docx]

**Additional file 7. List of 23 gene-based molecular markers and their syntenic gene annotations in rice genome.**

| **Barley Markers** | **Physical Position^*^** | **Rice Genes** | **Gene Annotations** |
| --- | --- | --- | --- |
| *Contig_41777* | 7460819 bp | *LOC_Os06g02180* | Cellulose synthase-like protein (*OsCSLD2*) |
| *Contig_49664* | 12616008 bp | *LOC_Os06g02780* | Aspartic protease |
| *Contig_45091* | 12947275 bp | *LOC_Os06g02960* | Uncharacterized protein |
| *Contig_2550647* | 12864976 bp | *LOC_Os06g03150* | Uncharacterized protein |
| *Contig_38877* | 12857350 bp | *LOC_Os06g03486* | Uncharacterized protein |
| *Contig_1574023-1* | 13261076 bp | *LOC_Os06g03530* | Pentatricopeptide repeat (PPR) protein |
| *Contig_1574023-2* | 13259453 bp | *LOC_Os06g03540* | Oligopeptide transporter (*OsOPT2*) |
| *Contig_136151* | 13624091 bp | *LOC_Os06g03580* | Zinc finger protein |
| *Contig_57240* | 13700379 bp | *LOC_Os06g03600* | SEUSS protein |
| *Contig_39163* | 13961363 bp | *LOC_Os06g03610* | Receptor-like protein kinase (RLK) |
| *Contig_37396* | 13817251 bp | *LOC_Os06g03630* | Uncharacterized protein |
| *Contig_7843* | 14293802 bp | *LOC_Os06g03660* | Peroxisomal membrane protein (PMP) |
| *Contig_61141* | 14789628 bp | *LOC_Os06g03670* | C-repeat binding protein (*OsCBF1*) |
| *Contig_405119* | 14772714 bp | *LOC_Os06g03682* | CDPK like protein |
| *Contig_57666* | 14801472 bp | *LOC_Os06g03690* | RNA recognition motif containing protein |
| *Contig_25551222* | 14944546 bp | *LOC_Os06g03710* | DELLA protein (*OsSLR1*) |
| *Contig_2179585* | 14944546 bp | *LOC_Os06g03710* | DELLA protein (*OsSLR1*) |
| *Contig_2551045* | Unknown | *LOC_Os06g03770* | Half ABC transporter(*OsABCB23*) |
| *SCRI_RS_139563* | 15857255 bp | *LOC_Os06g03790* | Ribosomal protein L47 |
| *SCRI_RS_235422* | 15923399 bp | *LOC_Os06g03850* | Impaired sucrose induction 1 |
| *Contig_49158* | 17089867 bp | *LOC_Os06g04200* | Granule-bound starch synthase (*GBSSⅠ*) |
| *SCRI_RS_158512* | 19927407 bp | *LOC_Os06g04300* | tRNA 2-phosphotransferase 1 |
| *Contig_56539* | 19480773 bp | *LOC_Os01g73220* | Peroxidase precursor |

**^*^**Physical positions are presented according to the available genomic information for Morex from 2017 [54].
