# Supplementary material for: A major QTL on chromosome 7HS controls the response of barley seedling to salt stress in the Nure × Tremois population
Source: BMC Genet. 2017 Aug 22;18:79. doi: 10.1186/s12863-017-0545-z (PMC5568257; doi:10.1186/s12863-017-0545-z)
Supplement: Supplementary file 8 — Allelic state of selected tolerant and susceptible NT lines for molecular markers linked to the major QTL on chromosome 7HS. “N” and “T” represent the alleles from the parent Nure and Tremois, respectively. (DOCX 15 kb) [file 12863_2017_545_MOESM8_ESM.docx]

**Additional file 8. Allelic state of selected tolerant and susceptible NT lines for molecular markers linked to the major QTL on chromosome 7HS.** “N” and “T” represent the alleles from the parent Nure and Tremois, respectively.

| **Group** | **DH Lines** | **Molecular Markers on 7HS** | | | | | |
| --- | --- | --- | --- | --- | --- | --- | --- |
|  |  | ***Bmag0206*** | ***Bmag0007*** | ***Contig_61141*** | ***Contig_2179585*** | ***Contig_405119*** | ***Contig_57666*** |
| Tolerant | NT022 | N | N | N | N | N | N |
|  | NT030 | N | N | N | N | N | N |
|  | NT071 | N | N | N | N | N | N |
|  | NT086 | N | N | N | N | N | N |
|  | NT124 | N | N | N | N | N | N |
|  | NT141 | N | N | N | N | N | N |
| Susceptible | NT043 | T | T | T | T | T | T |
|  | NT051 | T | T | T | T | T | T |
|  | NT084 | T | T | T | T | T | T |
|  | NT085 | T | T | T | T | T | T |
|  | NT098 | T | T | T | T | T | T |
|  | NT131 | T | T | T | T | T | T |
